# Psychological pathways linking digital museum environments to heritage-related cultural identity among generation Z college students in China

**DOI:** 10.3389/fpsyg.2026.1979445

**Published:** 2026-10-27

**Authors:** Bing Zhao, Wenbin Li, Xinlei Hou, Ting Huang

**Affiliations:** Southwest Minzu University, Chengdu, China

**Keywords:** cultural heritage understanding, digital museums, heritage-related cultural identity, media psychology, social presence, telepresence

## Abstract

**Introduction:**

Digital museums function as media environments whose communication and interface cues may be associated with heritage-related cultural identity. This study examined these associations through psychological pathways and configurational heterogeneity among Generation Z college students in China.

**Methods:**

Drawing on the Stimulus–Organism–Response framework, cross-sectional survey data from 425 students with prior experience using the Intelligent National Digital Museum were analyzed using partial least squares structural equation modeling (PLS-SEM) and fuzzy-set qualitative comparative analysis (fsQCA).

**Results:**

Social media marketing and website aesthetics were positively associated with cultural heritage understanding, telepresence, and social presence, which were positively associated with heritage-related cultural identity. Telepresence showed the numerically strongest association with identity. Cultural heritage understanding also exhibited indirect associations through both forms of presence, suggesting its potential role as a cognitive hub. All four sequential indirect associations were significant but small in the primary model; the two originating from website aesthetics did not meet the 0.05 significance threshold in the alternative model. Exploratory fsQCA identified six configurations associated with high heritage-related cultural identity, summarized as cognition–immersion, cognition–social media, and aesthetics-social presence patterns. In this sample, no single condition met the necessity criterion for high heritage-related cultural identity, and configurations associated with low identity were not simple inverses of those associated with high identity.

**Discussion:**

Together, the findings support cognitive, spatial, and social associations linking digital museum environments with heritage-related cultural identity, while showing that positive average associations coexist with configurational heterogeneity, equifinality, and asymmetry.

## 1 Introduction

Digital museums increasingly function as media environments through which cultural heritage is selected, framed, visualized, and socially circulated. Interactive interfaces, immersive presentations, and networked participation are shifting heritage communication from one-way display toward more contextualized and participatory experiences (Yang and Zhang, 2022). This shift is particularly consequential for Generation Z college students, whose information seeking, communication, and cultural participation are increasingly mediated by digital technologies. From a media-psychological perspective, the central question is therefore not only whether young people accept or continue to use a digital museum, but how communication and interface cues are attended to, interpreted, and experienced, and how these internal states are associated with identity-relevant outcomes.

Existing research has developed along three main lines. The first focuses on technology acceptance, perceived usefulness, satisfaction, immersive experience, revisit intention, recommendation intention, continuance intention, and other technological and behavioral outcomes (Abdul Aziz et al., 2024; Cheng et al., 2024; Khalil et al., 2024; Gong et al., 2025; Yang et al., 2026). The second has begun to examine the cultural and psychological outcomes of digital cultural heritage experiences by incorporating constructs such as cultural value and cultural identity into models explaining offline visitation or electronic word-of-mouth behavior (Meng and Dolah, 2025; Zhang et al., 2026; Wang and Meng, 2024). The third employs methods such as partial least squares structural equation modeling (PLS-SEM) and fuzzy-set qualitative comparative analysis (fsQCA) to investigate the configurational complexity underlying digital cultural experiences (Deng et al., 2026).

Despite this progress, three gaps remain from a media-psychological perspective. First, the positioning of the outcome variable remains limited. Although cultural identity has entered digital cultural heritage research, it is often treated as an intermediate variable linking media experience with subsequent behavior. Comparatively fewer studies have sought to explain heritage-related cultural identity itself as the focal psychological response. Second, the integration of internal processes remains insufficient. Existing studies have examined cultural value, immersion, affective value, and place attachment, but cultural heritage understanding, spatial presence, and social presence have rarely been integrated within one model as distinguishable cognitive, spatial, and social processes. The media-psychological sequence through which communication and interface cues become associated with cultural identity therefore remains underdeveloped. Third, configurational explanations remain limited. Existing research can identify whether particular media-environment cues and psychological states are positively associated with cultural identity on average, but such average associations do not establish that the same conditions must be jointly present across cases. High heritage-related cultural identity may be associated with different combinations of media-environment cues, cultural understanding, and spatial or social presence, while configurations associated with low heritage-related cultural identity may not simply mirror those associated with high heritage-related cultural identity. It therefore remains unclear whether heritage-related cultural identity is characterized by a relatively uniform media-psychological architecture or by configurational heterogeneity and asymmetry.

To address these gaps, this study uses the Stimulus–Organism–Response (SOR) framework (Mehrabian and Russell, 1974) to organize a media-effects process. Social media marketing and website aesthetics are conceptualized as two perceived media-environment stimuli, representing networked cultural communication and interface-based presentation, respectively. Cultural heritage understanding, telepresence, and social presence represent internal psychological states at the cognitive, spatial, and social levels, and heritage-related cultural identity is positioned as the focal response. This formulation treats the digital museum neither as a psychologically neutral delivery channel nor as a direct determinant of identity. Instead, it examines how media cues acquire psychological significance through users’ meaning-making and subjective experience.

Building on this framework, the study examines indirect associations linking social media marketing and website aesthetics with heritage-related cultural identity through cultural heritage understanding, telepresence, and social presence. It also examines sequential indirect associations in which the media stimuli are linked first to cultural heritage understanding, then to telepresence or social presence, and finally to heritage-related cultural identity. PLS-SEM is used to estimate average net associations and theory-specified indirect pathways, whereas fsQCA identifies combinations of media-environment and psychological conditions associated with membership in sets representing high and low heritage-related cultural identity. Together, the two methods address average associations, equifinality, and configurational asymmetry without treating cross-sectional patterns as demonstrated temporal causation.

Accordingly, this study addresses the following research questions:

RQ1. How are social media marketing and website aesthetics associated with cultural heritage understanding, telepresence, and social presence?

RQ2. How are these internal psychological states associated with heritage-related cultural identity, and through which specific and sequential indirect pathways are social media marketing and website aesthetics associated with heritage-related cultural identity?

RQ3. Does heritage-related cultural identity exhibit configurational heterogeneity and asymmetry, and which combinations of media-environment and psychological conditions are associated with membership in sets representing high and low heritage-related cultural identity?

This study makes three principal contributions. Theoretically, it contributes to media psychology by shifting the focal outcome in digital museum research from technology acceptance and subsequent behavioral intentions to heritage-related cultural identity. Within the SOR framework, it distinguishes cultural heritage understanding, telepresence, and social presence as cognitive, spatial, and social processes through which networked communication and interface cues become psychologically meaningful. Theoretically, the study further examines whether these average associations imply a uniform media-psychological architecture or whether heritage-related cultural identity is associated with configurationally heterogeneous and asymmetric combinations of conditions. Methodologically, the combined use of PLS-SEM and fsQCA enables these aggregate and configurational patterns to be examined within a common analytical framework.

## 2 Literature review

### 2.1 Heritage-related cultural identity in digital museum research

Cultural identity concerns the system of meanings through which individuals understand and value a culture and maintain a psychological connection to it. Contemporary psychological research increasingly conceptualizes cultural identity as a multidimensional and context-dependent psychological structure rather than as a simple affirmation of group membership (Manago and McKenzie, 2024). Research on cultural attachment further suggests that culture can become an object of psychological attachment, satisfy individuals’ need for belonging, and shape how they connect with cultural groups and cultural meanings (Liu et al., 2025). Participation in culturally meaningful practices can also help sustain cultural identity by providing individuals with a sense of cultural continuity, belonging, and emotional connection (Varma and Siromahov, 2023).

Although the source scales from which the items were adapted use the label “cultural identity” (Cleveland et al., 2016; Zhang et al., 2019; Song et al., 2025), the present study refers to the operationalized construct as “heritage-related cultural identity” throughout the manuscript to reflect its more specific measurement scope. It is measured through participants’ sense of pride in the history and culture presented by the digital museum, recognition of the importance of cultural heritage preservation, personal connection to the cultural heritage, and emotional attachment to it. These indicators capture an evaluative and affective relationship with the cultural heritage being presented, rather than the full multidimensional domain of cultural identity. They do not, however, encompass all possible dimensions of cultural identity, such as ethnic self-categorization, national group membership, language, cultural practices, or the centrality of cultural identity within the self-concept.

This operationalization is particularly appropriate for cultural heritage experiences. From the perspective of environmental psychology, historical places and representations of history and culture do not automatically acquire psychological significance solely because of their physical attributes. Their psychological relevance also depends on whether individuals can recognize their historical meaning, connect new information with prior knowledge, imagine historical events, experience a sense of emotional proximity to history, and link the represented past to socially shared memories (Ries and Schwan, 2023). Research on cultural attachment similarly indicates that cultural symbols and practices acquire stronger psychological significance when they provide individuals with a sense of belonging, security, and continuity (Liu et al., 2025). Heritage-related cultural identity in digital museum contexts can therefore be understood as a cognitive–affective relationship that develops between individuals and historically meaningful cultural content.

Existing research on digital museums and digitally mediated museum experiences can be broadly divided into three streams. The first centers on technology acceptance and service use, primarily examining how perceived usefulness, perceived ease of use, service quality, and perceived risk are associated with adoption and behavioral intentions concerning museum mobile guides, augmented reality, and intelligent applications (Kang et al., 2018; Carvajal-Trujillo et al., 2021; Zhang et al., 2026). The second shifts attention toward visitors’ evaluations of their experiences, emphasizing experiential variables such as website aesthetics, interactivity, immersion, authenticity, perceived value, satisfaction, and loyalty (Xia et al., 2024; Çiftçi and Çizel, 2024; Wan et al., 2026). The third examines how digital technologies participate in cultural meaning-making and shape the psychological connections between individuals and cultural heritage, addressing cultural identity, cultural attachment, place attachment, cultural communication, and public engagement (Wang and Meng, 2024; Chen, 2024; Deng et al., 2026).

Psychological research on virtual galleries has shown that individual differences in personality, aesthetic fluency, and aesthetic responsiveness are associated with online museum visitation behavior (Rodriguez-Boerwinkle and Silvia, 2024). Research on museum digitalization has further connected technological mediation and cultural symbolism with identity-related cognition and public engagement (Wang and Meng, 2024). Other cultural heritage studies have positioned cultural identity as a mediator between cultural experiences and cultural communication or have incorporated it into cognitive pathways leading to behavioral intentions (Chen, 2024; Deng et al., 2026).

These studies establish the importance of cultural identity in digital cultural heritage experiences. However, these studies commonly position cultural identity as a mediator or antecedent of cultural transmission, public engagement, or heritage-related behavioral intentions (Chen, 2024; Deng et al., 2026; Wang and Meng, 2024). Comparatively few studies have sought to explain cultural identity itself as the focal psychological response. Moreover, cognitive meaning-making, subjective spatial positioning, and the sense of social co-presence have rarely been incorporated simultaneously into a psychological process model in which cultural identity constitutes the principal outcome. By positioning heritage-related cultural identity as the outcome variable and distinguishing among cognitive, spatial-experiential, and social-experiential psychological states, the present study addresses both the outcome gap and the mechanism gap in the existing literature.

### 2.2 Digital museums as psychologically active media environments

Digital museums are not psychologically neutral containers of content; they are media environments constituted by communication formats, interface affordances, cultural symbols, and interactive structures. Media psychology examines how media use interacts with cognition, affect, perception, and behavior rather than treating media as channels that transmit information unchanged (Walter, 2024). Related research also conceptualizes social media as an identity-relevant environment in which individuals receive social feedback, explore meanings, and negotiate the self (Pérez-Torres, 2024). Whether a digital museum becomes psychologically relevant to identity therefore depends not only on technological accessibility, but also on how cultural cues are selected, presented, attended to, interpreted, and socially experienced.

In digital museums, this media-psychological process involves at least two complementary systems. First, official social media channels constitute a networked communication environment that can increase the visibility, repetition, timeliness, personalization, and social reach of cultural content. Research on identity-relevant content on social media suggests that users may experience stronger social connectedness when a platform appears responsive to personally relevant content, but also shows that technological personalization alone does not guarantee social connection (Taylor and Chen, 2024). In museum contexts, social media marketing activities have likewise been associated with social presence, perceived social support, and flow (Luo et al., 2026).

Second, website aesthetics constitutes an interface-based presentation environment that structures how users perceive, navigate, and explore digital cultural content. Research on online digital exhibitions has identified unity, novelty, interactivity, and immersion as core dimensions of website aesthetics (Xia et al., 2024). Psychological research on virtual galleries further indicates that users’ aesthetic responsiveness and artwork characteristics are associated with viewing time, movement, and engagement within virtual museums (Rodriguez-Boerwinkle and Silvia, 2024). The media-psychological relevance of website aesthetics therefore extends beyond visual appeal to how the interface directs attention, supports exploration, and organizes cultural information processing.

Accordingly, the present study treats the digital museum as a media environment whose psychological effects are conditional rather than as a direct source of identity change. Communication and interface cues may be associated with heritage-related cultural identity through users’ meaning-making and experiences of spatial or social presence. This formulation also bounds the inferences that can be drawn: cross-sectional data can test patterns of association among media characteristics, internal psychological states, and heritage-related cultural identity, but cannot establish that media exposure strengthens or changes identity over time.

This media-psychological positioning clarifies the questions addressed by the model: under what internal processing conditions does cultural information move from exposure to understanding; under what experiential conditions do digital representations create psychological spatial proximity; and under what conditions does social communication make other people and cultural communities psychologically perceptible? Cultural heritage understanding, telepresence, and social presence are therefore modeled as distinct internal psychological states through which networked communication and interface cues are associated with heritage-related cultural identity.

### 2.3 The Stimulus–Organism–Response framework and model specification

The Stimulus–Organism–Response (SOR) framework originated in environmental psychology and is used to explain how environmental cues become associated with subsequent responses through individuals’ internal psychological states (Mehrabian and Russell, 1974). Within this framework, stimuli refer to the external environmental inputs to which individuals are exposed. The organism encompasses the cognitive, affective, perceptual, and experiential states that arise as individuals attend to, interpret, and experience these inputs. Responses refer to the psychological, attitudinal, or behavioral outcomes associated with these internal states.

The psychological value of the SOR framework lies in its refusal to assume that environmental features act directly and uniformly on all individuals. Digital media cues acquire psychological significance through attention, interpretation, and subjective experience. This principle is consistent with media-psychological accounts of the interaction among media environments, cognition, affect, and behavior (Walter, 2024). Research on historical places likewise indicates that environmental characteristics become experiential only in relation to visitors’ prior knowledge, motivations, social backgrounds, and psychological processing (Ries and Schwan, 2023). The present study therefore uses SOR to organize a process linking media cues, internal psychological processing, and an identity-relevant response rather than treating technology as a direct determinant of heritage-related cultural identity.

The SOR framework has been applied to research on online consumption, social media, virtual environments, and digital exhibitions. However, the response variables in these studies have generally centered on technology adoption, satisfaction, loyalty, revisit intention, and other behavioral intentions. For example, research on digital exhibitions has examined website aesthetics in relation to perceived restorativeness, place attachment, and loyalty (Xia et al., 2024), whereas research on museum virtual reality has investigated the relationships of presence and authenticity with satisfaction and revisit intention (Choi and Nam, 2024). Although recent digital cultural heritage research has begun to incorporate cultural identity, it has primarily positioned cultural identity as a mediator in pathways leading to heritage-related behavioral intentions (Deng et al., 2026). Comparatively few studies have positioned cultural identity itself as the focal response while systematically distinguishing among cognitive, spatial, and social organismic states.

To address these gaps, the constructs in the present study were selected according to the psychological process through which digital cultural content enters individual experience and acquires identity-related meaning, rather than by simply combining variables commonly used in digital museum research. The two types of stimuli capture the networked communication environment in which cultural content circulates and the digital interface through which it is experienced. The three organismic states correspond to cognitive meaning-making, telepresence within the represented space, and social co-presence. Heritage-related cultural identity is positioned as the focal identity-related response to be explained. This configuration accounts for the complementarity of the environmental inputs, the distinct psychological functions of the internal states, and the alignment between the outcome variable and the research question. The specific definitions of the constructs and their hypothesized relationships are developed in the following section.

## 3 Research model and hypotheses

The constructs were selected to maintain conceptual–measurement alignment and distinct roles within the SOR framework. Social media marketing (SMM) and website aesthetics (WA) represent users’ perceptions of networked cultural communication and interface-based presentation, respectively (Kim and Ko, 2012; Xia et al., 2024). Cultural heritage understanding (CHU), telepresence (TPS), and social presence (SPS) capture cognitive meaning-making, spatial presence, and perceived social co-presence, while heritage-related cultural identity (CI) is the focal response.

The resulting model specifies a media-psychological sequence from communication and interface cues to cognitive, spatial, and social states and then to heritage-related cultural identity (Cummings and Wertz, 2022; Hartmann et al., 2016; Ries and Schwan, 2023). WA’s immersion dimension concerns attentional absorption, whereas TPS concerns the distinct spatial experience of ‘being there.’ The model is a parsimonious, theory-specified account rather than an exhaustive or temporally causal representation; measurement sources and higher-order specifications are reported in the Method (Figure 1).

**FIGURE 1:**
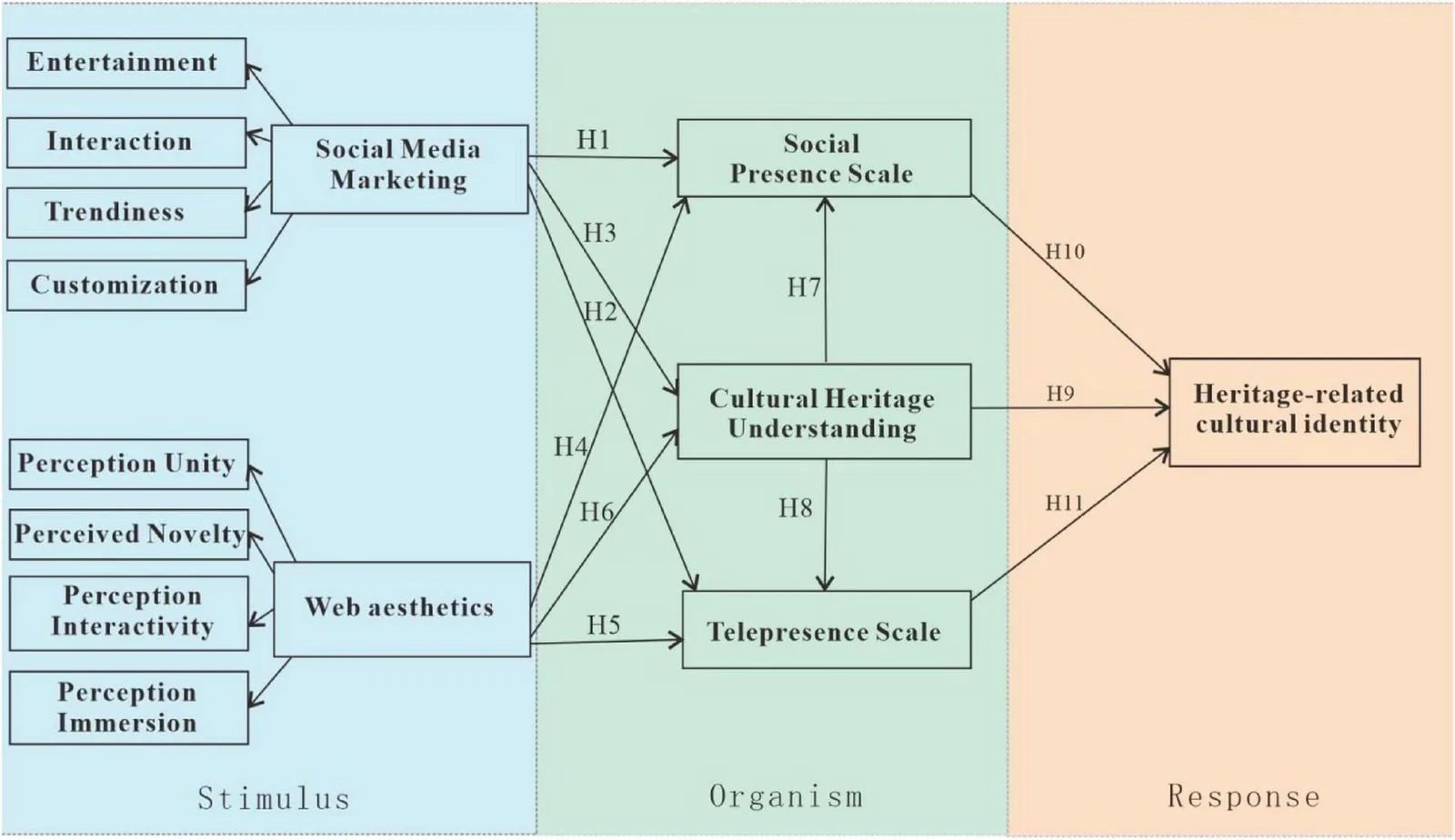
Conceptual model of the study.

### 3.1 Social media marketing as a networked communication stimulus

Social media marketing (SMM), as originally developed in marketing research, refers to organizational practices of content dissemination and participatory communication through social media platforms, which are associated with users’ cognition, attitudes, and behavior through interactive communication and networked circulation (Mangold and Faulds, 2009; Zeqiri et al., 2025). The construct is commonly conceptualized in terms of entertainment, interactivity, trendiness, and customization (Kim and Ko, 2012). The present study retains the scale label ‘social media marketing’ but does not treat it as a measure of promotional effectiveness. Instead, it represents users’ perceptions of networked cultural communication cues in the official social media channels of the Intelligent National Digital Museum.

This interpretation is based on the communicative functions represented by the four lower-order dimensions. Entertainment captures the capacity of social media content to attract and sustain attention; interaction reflects opportunities for reciprocal engagement with cultural information; trendiness captures the connection of heritage content with contemporary topics and circulating discourse; and customization reflects the perceived relevance of communication to users’ interests and informational needs. In the digital-museum context, these dimensions therefore describe how cultural content is made visible, engaging, timely, interactive, and personally relevant within a networked media environment, rather than how effectively a commercial product is promoted.

Social presence and telepresence constitute two experiential pathways through which such content may become psychologically engaging (Algharabat et al., 2018). Interactive and socially framed communication can increase the salience of museum personnel, other users, and culturally relevant groups, thereby strengthening users’ perceptions of the presence of others. Museum research has also shown that social media marketing activities are significantly associated with users’ social presence and perceived social support (Luo et al., 2026). Social media content may further strengthen telepresence by directing attention, evoking mental imagery, and transporting users into the world of a cultural narrative. Research on narrative transportation suggests that immersion based on attention, affect, and imagery enables mediated narratives to exert meaningful psychological effects (Green and Brock, 2000). Telepresence theory similarly defines this state as the subjective experience of “being there” in a mediated environment (Steuer, 1992). Recent tourism research further indicates that information quality and social network interaction can enhance a sense of spatial presence (Li et al., 2025). Accordingly:

H1: Social media marketing is positively associated with social presence.

H2: Social media marketing is positively associated with telepresence.

SMM may also facilitate cultural heritage understanding. Entertainment can attract attention, trendiness can connect cultural heritage with contemporary issues, customization can increase the personal relevance of the content, and interactivity enables users to explore or clarify cultural information. In museum and cultural tourism contexts, social media can expand users’ access to visual and interpretive content and help institutions communicate cultural narratives more effectively (Luo et al., 2026; Mele et al., 2021). Cultural psychology further suggests that culturally laden cues can activate relevant knowledge structures and influence how individuals construct meaning from subsequent information (Hong et al., 2000; Oyserman, 2011). SMM may therefore help users integrate fragmented content into a coherent understanding of the history, symbols, and values presented by the digital museum. Accordingly:

H3: Social media marketing is positively associated with cultural heritage understanding.

### 3.2 Website aesthetics as an interface-based media stimulus

Website aesthetics (WA) refers to users’ visual and experiential evaluations of a digital interface (Schenkman and Jönsson, 2000). Drawing on the website-aesthetics dimensions examined by Xia et al. (2024), this study models WA as a higher-order construct comprising perceived unity, novelty, interactivity, and immersion. Unity and novelty describe the coherence and freshness of the presentation, whereas interactivity and immersion reflect opportunities for exploration and attentional absorption. WA therefore represents users’ integrated perception of interface-based media cues rather than visual appeal alone or an objective design attribute. For this reason, perceived immersion is positioned within the stimulus component because it reflects users’ perception of the interface’s capacity to support attentional absorption, whereas telepresence is positioned within the organism component because it represents the internal spatial experience that may arise when users feel located within the mediated environment.

WA may be associated with social presence and telepresence through interrelated but distinct processes. Visual and social cues can increase the salience of other people, whereas interactive functions and timely feedback can make participation appear more responsive and reciprocal, thereby strengthening social presence (Park et al., 2021; Chen, 2023). Telepresence depends on whether users can construct a coherent representation of a mediated space and adopt that space as their primary spatial frame of reference (Wirth et al., 2007). Meta-analytic evidence further indicates that immersive technological affordances exert a substantial overall influence on users’ sense of presence (Cummings and Bailenson, 2016). Unity can help maintain environmental coherence, novelty can direct exploratory attention, interactivity can provide a sense of control, and immersion can sustain users’ engagement with the represented setting. WA and telepresence are therefore conceptually distinct: WA concerns users’ perceptions of interface attributes, whereas telepresence concerns the specific spatial experience of being present within the represented cultural environment. Accordingly:

H4: Website aesthetics is positively associated with social presence.

H5: Website aesthetics is positively associated with telepresence.

WA may also facilitate cultural heritage understanding by organizing attention and regulating cognitive effort. Processing fluency theory proposes that coherent and readily processed stimuli elicit more positive perceptual and affective responses (Reber et al., 2004). The pleasure–interest model further explains how novelty and moderate complexity can encourage deeper processing rather than merely produce visual preference (Graf and Landwehr, 2015). Before an evaluation is formed, aesthetic processing also involves stages such as classification, cognitive mastery, and interpretation (Leder et al., 2004). Consistent with these psychological perspectives, neurocognitive evidence indicates that webpage complexity and structural order influence the early allocation of attentional resources and subsequent information processing (Liu et al., 2022). A coherent structure may therefore help users recognize relationships between cultural artifacts and narratives, whereas novelty and interactivity may sustain the cognitive engagement required to understand these relationships. Accordingly:

H6: Website aesthetics is positively associated with cultural heritage understanding.

### 3.3 Cultural heritage understanding as a cognitive foundation

Cultural heritage understanding (CHU) refers to users’ understanding and psychological internalization of the history, symbols, narratives, and values presented by a digital museum (Bruner, 1996; Falk and Dierking, 2016). Cultural knowledge is not merely stored information. When culturally relevant representations become cognitively accessible and applicable to the current context, cultural knowledge can function as an interpretive framework (Hong et al., 2000; Oyserman, 2011). Virtual and interactive exhibitions can facilitate this process by situating cultural information within contextualized and experiential environments (Pujol and Champion, 2012; Wang and Meng, 2024; Choi and Nam, 2024). When users understand the relationships between cultural artifacts and narratives and their broader cultural contexts, an exhibition no longer appears as a collection of disconnected information but becomes a coherent system of meaning. Related evidence from research on college students’ art comprehension highlights the role of contextual information in connecting perceptual and emotional responses with symbolic and narrative interpretation (Wang et al., 2026).

Such understanding can provide a cognitive foundation for two forms of presence. Spatial presence theory proposes that users must first construct a mental model of a mediated environment before they can experience that environment as their primary spatial frame of reference (Wirth et al., 2007). Understanding the historical context and relationships among cultural symbols can make this mental model more coherent and facilitate psychological entry into the represented environment. CHU may also strengthen social presence because cultural objects embody the actions, relationships, and values of both historical and contemporary groups. When users understand these meanings, the people and social relationships embedded in heritage narratives may become more psychologically accessible, thereby providing a cognitive basis for social presence (Biocca et al., 2003; Triberti et al., 2025). Cultural familiarity may similarly influence whether a virtual environment is experienced as meaningful, immersive, and socially present (Triberti et al., 2025). Accordingly:

H7: Cultural heritage understanding is positively associated with social presence.

H8: Cultural heritage understanding is positively associated with telepresence.

CHU should also be directly associated with heritage-related cultural identity. Collective identification can encompass positive evaluation, perceived importance, shared meaning, a sense of connection, and emotional attachment (Ashmore et al., 2004). More specifically, social psychological research distinguishes group-level self-definition from self-investment, with the latter reflected in solidarity, satisfaction, and centrality (Leach et al., 2008). These components closely correspond to the pride, perceived importance of cultural heritage preservation, sense of connection, and emotional attachment measured by the cultural identity scale employed in this study. Understanding provides a cognitive foundation for such identification because individuals are unlikely to invest psychologically in cultural symbols whose meanings remain unclear. Recent research in digital cultural contexts indicates that cultural knowledge, knowledge acquisition, emotional connection, and psychological ownership are associated with cultural-identity outcomes (Xue et al., 2025; Jiang et al., 2026). Accordingly:

H9: Cultural heritage understanding is positively associated with heritage-related cultural identity.

### 3.4 Presence as an experiential pathway to heritage-related cultural identity

In the present study, heritage-related cultural identity is conceptualized as heritage-related cognitive–affective identification rather than as a comprehensive measure of ethnic identity, national identity, personal identity, or digital identity. Social presence and telepresence provide two complementary experiential conditions through which cultural meanings that have already been understood may acquire greater personal psychological relevance.

Social presence situates cultural heritage within a shared social frame. When communication partners are perceived as psychologically present during interactions associated with the digital museum, users may experience cultural interpretation as participation in collective relationships rather than as the solitary acquisition of information. This mechanism is consistent with the fundamental human motivation to form meaningful and enduring social bonds, which influences both cognition and affect (Baumeister and Leary, 1995). Social presence may therefore make the shared and relational character of cultural heritage more salient, providing a plausible basis for belonging and emotional attachment (Baumeister and Leary, 1995; Leach et al., 2008). More broadly, digital-media research identifies social engagement as one mechanism through which media use is associated with cultural-identity construction (Yao, 2025).

Telepresence, by contrast, provides a spatial-experiential pathway by reducing the subjective distance between users and culturally meaningful settings. Experiencing a cultural heritage environment as psychologically close can make its meanings more concrete and increase their relevance to the self. Research on place conceptualizes attachment and identity as interrelated cognitive–affective bonds involving self-extension, congruity, and the physical and social meanings of place (Droseltis and Vignoles, 2010; Scannell and Gifford, 2010). Digital heritage research similarly indicates that presence can connect physically distant cultural content with users’ experiences and evaluations (Zhang and Liu, 2026). Accordingly:

H10: Social presence is positively associated with heritage-related cultural identity.

H11: Telepresence is positively associated with heritage-related cultural identity.

### 3.5 Indirect and sequential psychological pathways

SOR predicts that media stimuli become associated with responses through internal states. Following mediation and multiple-mediator logic (MacKinnon et al., 2007; Preacher and Hayes, 2008), SMM and WA may therefore be indirectly associated with heritage-related cultural identity through CHU, telepresence, and social presence. CHU captures meaning construction, whereas the two forms of presence capture spatial and social immediacy; the three mechanisms are tested as distinct specific indirect pathways. Accordingly:

H12a: Social media marketing has a positive indirect association with heritage-related cultural identity through cultural heritage understanding.

H12b: Social media marketing has a positive indirect association with heritage-related cultural identity through telepresence.

H12c: Social media marketing has a positive indirect association with heritage-related cultural identity through social presence.

H13a: Website aesthetics has a positive indirect association with heritage-related cultural identity through cultural heritage understanding.

H13b: Website aesthetics has a positive indirect association with heritage-related cultural identity through telepresence.

H13c: Website aesthetics has a positive indirect association with heritage-related cultural identity through social presence.

The association between cultural heritage understanding and heritage-related cultural identity may itself operate through presence. Understanding relations among artifacts, settings, actors, and practices can make the mediated cultural environment more coherent, supporting the spatial mental model underlying telepresence and the psychological accessibility of others underlying social presence (Biocca et al., 2003; Wirth et al., 2007). These forms of immediacy can make understood cultural meanings more self-relevant, consistent with research on identification and place attachment (Leach et al., 2008; Scannell and Gifford, 2010). Accordingly:

H14a: Cultural heritage understanding has a positive indirect association with heritage-related cultural identity through telepresence.

H14b: Cultural heritage understanding has a positive indirect association with heritage-related cultural identity through social presence.

Finally, the theory-specified sequential model places CHU before telepresence or social presence because meaning construction is theorized to provide the context in which spatial and social immediacy can emerge. SMM and WA may first make cultural information accessible and coherent; understood meanings can then be organized into a spatial mental model or a social-relational frame, which may connect them with pride, personal connection, and attachment. The CHU–telepresence and CHU–social-presence sequences therefore represent cognitive–spatial and cognitive–social pathways to heritage-related cultural identity. Accordingly:

H15a: Social media marketing has a positive sequential indirect association with heritage-related cultural identity through cultural heritage understanding followed by telepresence.

H15b: Social media marketing has a positive sequential indirect association with heritage-related cultural identity through cultural heritage understanding followed by social presence.

H15c: Website aesthetics has a positive sequential indirect association with heritage-related cultural identity through cultural heritage understanding followed by telepresence.

H15d: Website aesthetics has a positive sequential indirect association with heritage-related cultural identity through cultural heritage understanding followed by social presence.

## 4 Method

The study protocol was approved by the Ethics Committee of Southwest Minzu University on September 11, 2025 (Approval No. SMU-202501162), and all participants provided electronic informed consent before participation. Full ethics and consent statements are reported at the end of the manuscript.

### 4.1 Digital museum context

This study used the Intelligent National Digital Museum (https://www.chnmuseum.cn), a national-level Chinese platform integrating digitized heritage resources, online exhibitions, intelligent guides, and multimedia interaction. Its thematically distinct modules allow users to select exhibitions according to their interests; the established platform name is retained to identify the empirical media context accurately.

One representative example is the Joint Exhibition of Cultural Relics from the Palace Museum and the National Museum of China, which brings together more than 400 valuable artifacts from the two institutions. Through high-precision 3D modeling and integrated visual and textual presentation, the exhibition digitally reconstructs and presents the collections, facilitates the integration of cultural resources across museums, and provides users with a more immersive online viewing experience.

Before the formal survey, participants were provided with the website address and introduced to several representative exhibitions through images and textual descriptions (see Table 1). These materials served only to standardize the survey referent and ensure that respondents understood which platform the questionnaire concerned; they did not constitute an experimental manipulation and did not replace the requirement that participants have prior experience using the platform.

**TABLE 1:** Representative digital exhibitions within the Intelligent National Digital Museum.

No.	Digital exhibition theme	Exhibition title	Brief description
01	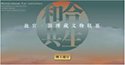	Joint Exhibition of Cultural Relics from the Palace Museum and the National Museum of China	The exhibition features over 400 valuable artifacts from the Palace Museum and the National Museum of China. By employing high-precision 3D modeling and the integration of visual and textual materials, the artifacts are digitally presented, facilitating the integration of cultural resources across museums and offering an immersive online exhibition experience.
02	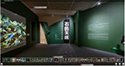	The Natural Charm of Stone	The exhibition features more than 160 ornamental stones, presenting their diverse forms and cultural meanings through four thematic sections. Using high-precision 3D modeling and the integration of images and textual information, the collections are digitally displayed. Visitors can click to view photographs of the physical objects and participate in online comment interactions, enhancing engagement in the viewing experience.
03	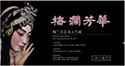	The Artistic Legacy of Mei Lanfang	Taking the artistic life of Mei Lanfang as the main narrative thread, the exhibition combines precious cultural relics, archival materials, and panoramic imaging technology to present his artistic creations across different periods and the development of the Mei school of Peking Opera.
04	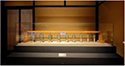	Harmony of Heaven and Earth: Musical Instruments Exhibition	The exhibition brings together more than 200 precious ancient Chinese musical instruments. Through the integration of 3D modeling and high-definition images, the forms and details of the artifacts are digitally presented, systematically illustrating the development of Chinese music and the cultural spirit of ritual and music traditions.
05	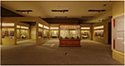	Carving the Universe: Chaozhou Woodcarving Exhibition	The exhibition showcases more than 160 representative works of Chaozhou woodcarving, including classic artifacts from the Ming and Qing dynasties to the modern period, as well as works by contemporary masters. Through the integration of 3D modeling and images, it presents the carving techniques, thematic meanings, and folk aesthetic characteristics embodied in the artworks.

Source of images: Official website of the National Museum of China (http://www.chnmuseum.cn). Reproduced with permission.

The exhibitions presented in Table 1 are representative digital exhibitions available within the same platform, although they cover different themes and may involve different contributing institutions or cultural subjects.

### 4.2 Research design

All measurement items used in this study were adapted and contextualized from previously published scales (Jiang et al., 2016; Vashisht and Chauhan, 2017; Tcha-Tokey et al., 2016; Xia et al., 2024; Kim and Ko, 2012; Liu et al., 2023; Çil et al., 2023; Wang and Meng, 2024; Krasonikolakis et al., 2018; Zhu et al., 2025; Alalwan et al., 2025; Kreijns et al., 2011; Alsayer and Lowenthal, 2025; Cleveland et al., 2016; Zhang et al., 2019; Song et al., 2025). All items were measured using a seven-point Likert scale ranging from 1 (strongly disagree) to 7 (strongly agree). The complete wording and sources are provided in Supplementary Table 1.

The questionnaire consisted of three sections. The first section presented the study information, an introduction to the Intelligent National Digital Museum, informed consent, and two eligibility questions concerning participants’ prior use of the platform and prior exposure to content disseminated through its official social media channels. Only respondents who provided informed consent and confirmed both prior platform use and prior exposure to the official social media content were allowed to proceed to the formal questionnaire; otherwise, the survey was automatically terminated. Items assessing website aesthetics, cultural heritage understanding, telepresence, social presence, and heritage-related cultural identity referred to participants’ overall experience with the platform rather than to any single exhibition listed in Table 1, whereas the social media marketing items specifically referred to participants’ experience of the platform’s official social media communication. The second section collected participants’ demographic information. The third section contained the formal measurement items.

Because the original measurement items were developed in English, a professional English-language instructor first translated them into Chinese. A second instructor then back-translated the Chinese version into English, following the back-translation procedure described by Brislin (1970), to assess semantic consistency and accuracy.

Prior to the formal survey, five experts reviewed the questionnaire for semantic clarity, redundancy, contextual appropriateness, and conceptual coverage. Based on their feedback, nine items that were considered repetitive, ambiguous, or insufficiently suited to the digital museum context were removed. In making these revisions, care was taken to ensure that the retained items continued to represent the core conceptual domain of each construct. A pilot test was subsequently conducted with 100 valid responses. The pilot test showed that one item had an outer loading below 0.50. After considering its statistical performance and conceptual relevance, the item was removed, resulting in the final questionnaire used for the formal survey.

### 4.3 Data collection

This study targeted Generation Z college students aged between 18 and 26 who had prior experience using the Intelligent National Digital Museum and had been exposed to content disseminated through its official social media channels. Data were collected through an anonymous online questionnaire hosted on Wenjuanxing, with the survey conducted from January 1 to January 31, 2026.

Following approaches used in prior research, the questionnaire was initially distributed through voluntary sampling, whereby the survey link was shared on social media platforms (Shahzad et al., 2025). Subsequently, a snowball sampling method was employed: members of the research team distributed the questionnaire to university instructors they knew at different institutions and asked them to further share the survey link through their social networks (Ivanov et al., 2024; Shahzad et al., 2025). In total, 506 students from universities across different regions of China participated in the survey.

Of the 506 recorded responses, only cases confirming prior platform use and meeting the data-quality criteria were retained. Incomplete responses, unusually fast completions, and invariant response patterns were excluded (Meade and Craig, 2012), yielding 425 valid cases for analysis. The demographic characteristics of the respondents are presented in Table 2.

**TABLE 2:** Demographic characteristics of respondents.

Demographic factors	Category	Number	Percent (%)
Gender	Male	214	50.4
	Female	211	49.6
Academic Year	Freshman	97	22.8
	Sophomore	74	17.4
	Junior	137	32.2
	Senior	117	27.5
Major Category	Science and Engineering	215	50.6
	Humanities and Social Sciences	170	40.0
	Art and Design	40	9.4
Use of National Digital Museum		425	100.0

Sample adequacy was assessed using the inverse square root method for PLS-SEM (Kock and Hadaya, 2018). With a significance level of 0.05, statistical power of 0.80, and a minimum expected path coefficient of 0.15, the estimated minimum sample size was 275. The final sample of 425 therefore exceeded this benchmark.

### 4.4 Data analysis

This study employed a cross-sectional design and combined partial least squares structural equation modeling (PLS-SEM) with fuzzy-set qualitative comparative analysis (fsQCA) to examine average variable associations and configurations of conditions related to heritage-related cultural identity. PLS-SEM is suitable for estimating complex models involving multiple latent variables and indirect pathways and does not require multivariate normality (Hair et al., 2019). Structural-path significance was assessed using 5,000 bootstrap resamples. Given the cross-sectional design, structural coefficients and indirect effects are interpreted as theory-specified associations rather than causally ordered effects.

Social media marketing (SMM) and website aesthetics (WA) were specified as reflective–reflective higher-order constructs. In this study, social media marketing and website aesthetics were conceptualized as users’ global perceptual evaluations of the platform’s communication activities and digital interface experience, rather than as indices formed by aggregating independently weighted objective features. Accordingly, the SOR “stimuli” in this study should be understood as retrospectively perceived media-environment cues rather than independently observed or experimentally manipulated environmental features. At the perceptual level, the overall evaluation of social media marketing is manifested through users’ perceptions of entertainment, interaction, trendiness, and customization, whereas the overall evaluation of website aesthetics is manifested through perceived unity, novelty, interactivity, and immersion. Although the lower-order dimensions retain conceptually distinct content, they refer to the same platform and evaluative target and are treated as domain-specific manifestations of the corresponding higher-order perception. Accordingly, both constructs were specified as reflective–reflective higher-order models (Chen and Lin, 2019; Sarstedt et al., 2019).

As a complementary configurational method, fsQCA was used to identify multiple sufficient combinations associated with membership in sets representing high or low heritage-related cultural identity (Shahzad et al., 2025). PLS-SEM examined average net associations and specific indirect effects, whereas fsQCA revealed alternative combinations of conditions. Here, sufficiency denotes a set-theoretic relation within the sample and should not be interpreted as demonstrated temporal causation.

## 5 Results

### 5.1 Symmetric analysis

#### 5.1.1 Measurement model assessment

Reliability and validity were satisfactory (Tables 3, 4). All Cronbach’s α and composite-reliability values exceeded 0.70, all item loadings exceeded 0.70, and all AVE values exceeded 0.50, supporting internal consistency and convergent validity (Fornell and Larcker, 1981; Hair et al., 2019). The Fornell–Larcker criterion was met and every HTMT value was below 0.85, supporting discriminant validity (Henseler et al., 2015).

**TABLE 3:** Assessment of the reflective lower-order measurement models.

Construct	Items	Factor loading	Cronbach’s α	CR	AVE
Perception unity	PU1	0.930	0.928	0.954	0.874
	PU2	0.940			
	PU3	0.935			
Perceived novelty	PN1	0.922	0.920	0.950	0.863
	PN2	0.934			
	PN3	0.930			
Perception interactivity	PI1	0.934	0.926	0.953	0.870
	PI2	0.929			
	PI3	0.936			
Perception immersion	PIM1	0.923	0.921	0.950	0.863
	PIM2	0.924			
	PIM3	0.940			
Entertainment	ENT1	0.933	0.932	0.957	0.881
	ENT2	0.936			
	ENT3	0.947			
Interaction	INT1	0.932	0.927	0.954	0.873
	INT2	0.935			
	INT3	0.935			
Trendiness	TRE1	0.935	0.927	0.954	0.873
	TRE2	0.937			
	TRE3	0.931			
Customization	CUS1	0.935	0.930	0.955	0.877
	CUS2	0.939			
	CUS3	0.936			
Cultural Heritage Understanding	CHU1	0.920	0.895	0.934	0.826
	CHU2	0.890			
	CHU3	0.916			
Telepresence Scale	TPS1	0.921	0.893	0.933	0.824
	TPS2	0.890			
	TPS3	0.912			
Social Presence Scale	SPS1	0.882	0.891	0.932	0.821
	SPS2	0.914			
	SPS3	0.922			
Heritage-related cultural identity	CI1	0.910	0.920	0.943	0.806
	CI2	0.904			
	CI3	0.888			
	CI4	0.889			

CR, composite reliability; AVE, average variance extracted. PU, PN, PI, and PIM represent the lower-order components of WA, whereas ENT, INT, TRE, and CUS represent the lower-order components of SMM.

**TABLE 4:** Discriminant validity.

Fornell – Larcker Criterion												
	CHU	CI	CUS	ENT	INT	PI	PIM	PN	PU	SPS	TPS	TRE
CHU	**0.909**	**0.898**	**0.937**	**0.939**	**0.934**	**0.933**	**0.929**	**0.929**	**0.935**	**0.906**	**0.908**	**0.934**
CI	0.356											
CUS	0.292	0.255										
ENT	0.258	0.247	0.483									
INT	0.259	0.282	0.490	0.484								
PI	0.162	0.199	0.179	0.115	0.204							
PIM	0.184	0.267	0.164	0.188	0.180	0.492						
PN	0.229	0.249	0.250	0.185	0.206	0.490	0.482					
PU	0.198	0.233	0.192	0.165	0.223	0.463	0.477	0.484				
SPS	0.376	0.351	0.271	0.245	0.263	0.180	0.208	0.210	0.198			
TPS	0.318	0.366	0.240	0.199	0.249	0.211	0.206	0.206	0.224	0.341		
TRE	0.194	0.239	0.497	0.509	0.515	0.122	0.097	0.105	0.114	0.231	0.208	
Heterotrait-monotrait ratio												
CHU												
CI	0.389	0.274	0.518	0.520	0.220	0.532	0.523	0.523	0.216	0.382	0.228	
CUS	0.319											
ENT	0.279	0.267										
INT	0.283	0.306	0.527									
PI	0.177	0.216	0.193	0.124								
PIM	0.202	0.292	0.177	0.202	0.195							
PN	0.251	0.272	0.270	0.199	0.223	0.530						
PU	0.215	0.252	0.206	0.178	0.241	0.498	0.516					
SPS	0.422	0.387	0.296	0.268	0.288	0.197	0.229	0.231				
TPS	0.354	0.401	0.262	0.217	0.273	0.233	0.227	0.227	0.247			
TRE	0.212	0.259	0.534	0.547	0.554	0.133	0.105	0.114	0.123	0.253		

CHU, cultural heritage understanding; CI, heritage-related cultural identity; CUS, customization; ENT, entertainment; INT, interaction; PI, perception interactivity; PIM, perception immersion; PN, perceived novelty; PU, perception unity; SPS, Social Presence Scale; TPS, Telepresence Scale; TRE, Trendiness. Values on diagonal, which are bold, are the square root of AVE At the higher-order level, all SMM and WA dimension loadings exceeded 0.70 and were significant at *p* < 0.001. Both higher-order constructs also met the reliability and convergent-validity criteria (Table 5).

**TABLE 5:** Assessment of the reflective-reflective higher-order constructs.

HOC	LOC	Loading	Mean	SD	*t*	*p*	α	ρ_A_	ρ_C_	AVE
SMM	Customization	0.784	0.783	0.026	30.266	<0.001	0.924	0.924	0.935	0.545
	Entertainment	0.784	0.784	0.027	29.290	<0.001				
	Interaction	0.789	0.789	0.026	29.996	<0.001				
	Trendiness	0.798	0.798	0.025	31.758	<0.001				
WA	Perceived interactivity	0.782	0.781	0.027	28.986	<0.001	0.919	0.919	0.931	0.530
	Perceived immersion	0.783	0.784	0.027	28.546	<0.001				
	Perceived novelty	0.786	0.787	0.027	29.575	<0.001				
	Perceived unity	0.775	0.775	0.028	27.692	<0.001				

HOC, higher-order construct; LOC, lower-order component; SD, bootstrap standard deviation; SMM, social media marketing; WA, website aesthetics; α, Cronbach’s alpha; ρ_A_, rho_A reliability coefficient; ρ_C_, composite reliability. The constructs were estimated as reflective-reflective higher-order models using the repeated indicators approach. All p values reported as 0.000 by SmartPLS are presented as *p* < 0.001. The HOC–LOC loadings represent the relationships between each higher-order construct and its lower-order components, whereas α, ρ_A_, ρ_C_, and AVE were calculated from the repeated indicators assigned to the higher-order construct. Therefore, the reported AVE is not the arithmetic mean of the squared HOC–LOC loadings.

At the structural-model level, HTMT values ranged from 0.271 to 0.422, below 0.85, supporting the distinctiveness of SMM, WA, CHU, SPS, TPS, and CI (Table 6).

**TABLE 6:** Discriminant validity of the structural-model constructs based on HTMT.

Construct	CHU	CI	SMM	SPS	TPS	WA
CHU	–	–	–	–	–	–
CI	0.389					
SMM	0.348	0.352				
SPS	0.422	0.387	0.351			
TPS	0.354	0.401	0.312	0.382		
WA	0.271	0.331	0.296	0.280	0.299	

CHU, cultural heritage understanding; CI, heritage-related cultural identity; SMM, social media marketing; SPS, social presence; TPS, telepresence; WA, website aesthetics.

#### 5.1.2 Structural model assessment

##### 5.1.2.1 Direct effects

The structural-model VIF values ranged from 1.080 to 1.244, below the recommended threshold of 3, indicating no serious multicollinearity (Hair et al., 2019). Bootstrapping with 5,000 resamples showed that all direct paths (H1–H11) were positive and statistically significant: SMM and WA were associated with CHU, telepresence, and social presence, and all three organism states were associated with heritage-related cultural identity (Figure 2 and Table 7).

**FIGURE 2:**
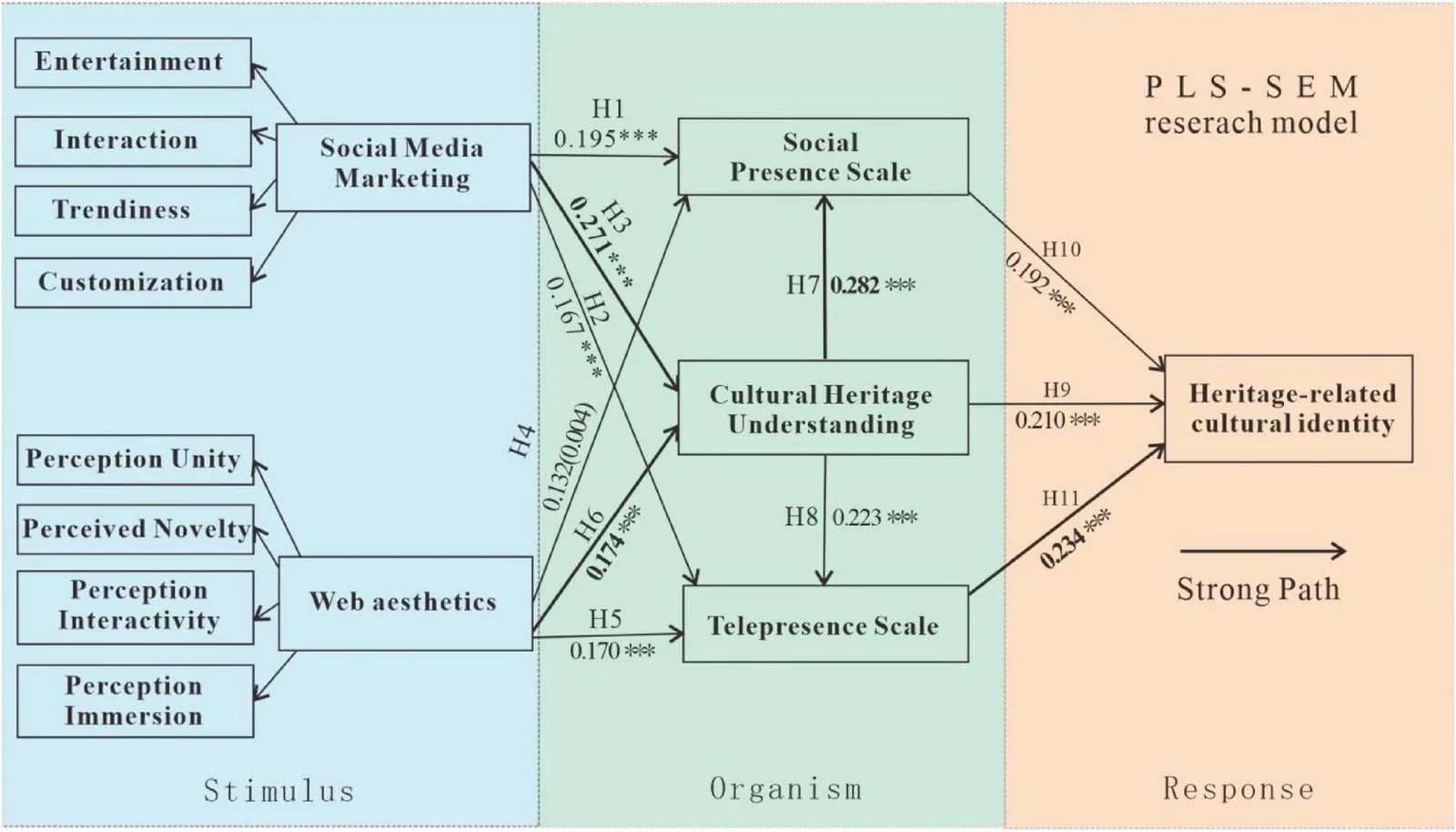
Results of the structural model based on the S–O–R framework.

**TABLE 7:** Explanatory power and predictive relevance.

H	Relationship	Coefficient	Std. Dev.	*t*-value	f^2^	VIF	*p*-value	Supported
H1	SMM → SPS	0.195	0.050	3.879	0.041	1.165	<0.001	Yes
H2	SMM → TPS	0.167	0.049	3.408	0.029	1.165	0.001	Yes
H3	SMM→ CHU	0.271	0.054	5.043	0.078	1.080	<0.001	Yes
H4	WA → SPS	0.132	0.045	2.911	0.019	1.115	0.004	Yes
H5	WA → TPS	0.170	0.050	3.435	0.031	1.115	0.001	Yes
H6	WA → CHU	0.174	0.053	3.297	0.032	1.080	0.001	Yes
H7	CHU → SPS	0.282	0.053	5.298	0.087	1.148	<0.001	Yes
H8	CHU→TPS	0.223	0.048	4.660	0.052	1.148	<0.001	Yes
H9	CHU→CI	0.210	0.053	3.967	0.047	1.223	<0.001	Yes
H10	SPS → CI	0.192	0.052	3.667	0.038	1.244	<0.001	Yes
H11	TPS→ CI	0.234	0.054	4.287	0.059	1.188	<0.001	Yes
					**Q^2^**	**R^2^**		
				SPS	0.120	0.196		
				CHU	0.117	0.125		
				TPS	0.110	0.159		
				CI	0.122	0.222		

The model explained 12.5% of the variance in CHU, 19.6% in social presence, 15.9% in telepresence, and 22.2% in heritage-related cultural identity, indicating meaningful but modest explanatory power.

##### 5.1.2.2 Indirect effects

In the primary model, all 12 hypothesized indirect effects were statistically significant because their 95% bias-corrected bootstrap confidence intervals excluded zero (Table 8). SMM and WA were each indirectly associated with heritage-related cultural identity through CHU, telepresence, and social presence, supporting H12a–H13c. The largest specific indirect association from SMM was through CHU (β = 0.057), whereas the largest from WA was through telepresence (β = 0.040). CHU was also indirectly associated with heritage-related cultural identity through telepresence (β = 0.052) and social presence (β = 0.054), supporting H14a–H14b. The four sequential paths supported H15a–H15d in the primary model, but their magnitudes were small (β = 0.009–0.015). The alternative-model analysis reported below further showed that the two sequential associations originating from WA did not meet the 0.05 significance threshold when direct stimulus–identity paths were included. Their statistical support therefore depended on model specification.

**TABLE 8:** Specific and sequential indirect effects in the primary structural model.

Hypothesis	Indirect path	Coefficient (β)	Std. Dev.	*t*-value	*p*-value	95% BC Bootstrap CI	Decision
H12a	SMM → CHU → CI	0.057	0.019	2.919	0.004	[0.026, 0.103]	Supported
H12b	SMM → TPS → CI	0.039	0.016	2.507	0.012	[0.015, 0.077]	Supported
H12c	SMM → SPS → CI	0.037	0.017	2.219	0.027	[0.012, 0.079]	Supported
H13a	WA → CHU → CI	0.036	0.016	2.337	0.019	[0.012, 0.075]	Supported
H13b	WA → TPS → CI	0.040	0.016	2.527	0.012	[0.016, 0.078]	Supported
H13c	WA → SPS → CI	0.025	0.012	2.074	0.038	[0.007, 0.056]	Supported
H14a	CHU → TPS → CI	0.052	0.017	3.129	0.002	[0.025, 0.092]	Supported
H14b	CHU → SPS → CI	0.054	0.017	3.117	0.002	[0.025, 0.094]	Supported
H15a	SMM → CHU → TPS → CI	0.014	0.006	2.514	0.012	[0.006, 0.028]	Supported
H15b	SMM → CHU → SPS → CI	0.015	0.006	2.507	0.012	[0.006, 0.030]	Supported
H15c	WA → CHU → TPS → CI	0.009	0.004	2.156	0.031	[0.003, 0.020]	Supported
H15d	WA → CHU → SPS → CI	0.009	0.004	2.102	0.036	[0.003, 0.022]	Supported

SMM, social media marketing; WA, website aesthetics; CHU, cultural heritage understanding; TPS, telepresence; SPS, social presence; CI, heritage-related cultural identity; β, standardized indirect effect; Std. Dev., bootstrap standard deviation; BC, bias-corrected. The 95% bias-corrected bootstrap confidence intervals were estimated using 5,000 subsamples. An indirect effect was considered statistically significant when the corresponding confidence interval did not include zero.

##### 5.1.2.3 Alternative structural model robustness check

As a robustness check, an alternative structural model was estimated by adding direct paths from social media marketing and website aesthetics to heritage-related cultural identity while retaining all relationships specified in the primary model (Table 9). The purpose of this analysis was to examine whether the principal organism-level and indirect associations remained stable when direct stimulus–response relationships were additionally allowed. Both added direct paths were statistically significant (SMM → CI: β = 0.135, *p* = 0.011; WA → CI: β = 0.139, *p* = 0.007). The explained variance in heritage-related cultural identity increased from R^2^ = 0.222 in the primary model to R^2^ = 0.264 in the alternative model. Most of the principal specific indirect associations remained statistically significant, including the pathways from SMM and WA through cultural heritage understanding and telepresence, as well as the indirect pathways from cultural heritage understanding through telepresence and social presence. The two weaker sequential indirect paths originating from website aesthetics, however, no longer reached the conventional 0.05 significance threshold (WA → CHU → TPS → CI: β = 0.007, *p* = 0.052; WA → CHU → SPS → CI: β = 0.007, *p* = 0.061). Overall, the alternative specification supports the coexistence of direct and indirect associations. However, the two WA-originating sequential associations were smaller and no longer met the 0.05 significance threshold, indicating greater sensitivity to model specification.

**TABLE 9:** Robustness results from the alternative structural model.

Alternative-model result	β / R^2^	*P*
SMM → CI	0.135	0.011
WA → CI	0.139	0.007
CI R^2^	0.264	–
WA → CHU → TPS → CI	0.007	0.052
WA → CHU → SPS → CI	0.007	0.061

### 5.2 Asymmetric analysis

#### 5.2.1 Data calibration

Before the fsQCA analysis, the raw seven-point Likert-scale data were transformed into fuzzy-set membership scores using the direct calibration method, following previous studies (Wang et al., 2025). The 95th, 50th, and 5th percentiles of each construct were used as the anchors for full membership, the crossover point, and full non-membership, respectively. The calibrated scores, ranging from 0 to 1, were generated in fsQCA 4.0 and used in the subsequent asymmetric analysis (see Table 10). To avoid retaining cases exactly at the maximum-ambiguity crossover, calibrated scores of 0.50 were adjusted to 0.5001 before truth-table construction.

**TABLE 10:** Descriptive statistics of the variables (after calibration).

Construct	Full membership (95thpercentile)	Crossoverpoint (50thpercentile)	Fullnon-membership (5thpercentile)	Mean (SD)	Min	Max	N
Website aesthetics	6.5000	5.3333	2.4167	0.59 (0.32)	0.04	0.97	425
Social media marketing	6.5000	5.3333	2.2667	0.60 (0.32)	0.04	0.97	425
Cultural heritage understanding	6.6667	6.0000	2.3333	0.55 (0.31)	0.02	0.99	425
Telepresence Scale	6.6667	6.0000	2.3333	0.55 (0.32)	0.02	0.99	425
Social Presence Scale	6.6667	6.0000	2.0000	0.56 (0.30)	0.02	0.99	425
Heritage-related cultural identity	6.7500	6.0000	2.2500	0.54 (0.29)	0.02	0.98	425

#### 5.2.2 Necessity analysis

After calibration, a necessity analysis was conducted to determine whether any single antecedent condition was necessary for the outcome (Dul, 2016). As shown in Table 11, no condition reached the recommended necessity-consistency threshold of 0.90 (Foroughi et al., 2024). Thus, no single media-environment or psychological condition was universally necessary for membership in the set representing high heritage-related cultural identity, supporting a configurational interpretation.

**TABLE 11:** Necessity analysis for high and low heritage-related cultural identity.

Antecedent conditions	High heritage-related cultural identity		Low heritage-related cultural identity	
	Consistency	Coverage	Consistency	Coverage
Website aesthetics	0.77	0.71	0.69	0.54
∼ Website aesthetics	0.50	0.66	0.63	0.70
Social media marketing	0.78	0.71	0.70	0.54
∼ Social media marketing	0.49	0.66	0.62	0.71
Cultural heritage understanding	0.75	0.73	0.68	0.56
∼ Cultural heritage understanding	0.55	0.66	0.67	0.69
Telepresence Scale	0.74	0.72	0.67	0.56
∼ Telepresence Scale	0.55	0.66	0.66	0.69
Social Presence Scale	0.77	0.75	0.67	0.56
∼ Social Presence Scale	0.54	0.66	0.69	0.72

#### 5.2.3 Configurational analysis

After data calibration and necessity analysis, fsQCA 4.0 was used to identify configurations. The intermediate solution served as the main basis for interpretation. For high heritage-related cultural identity, the presence of all five antecedent conditions was specified as the directional expectation because the theoretical model proposed positive associations. For low heritage-related cultural identity, the absence of these conditions was specified because lower levels of the media-environment and psychological conditions were expected to be associated with lower identity. These expectations guided counterfactual simplification; they did not require the low-identity configurations to mirror the high-identity configurations. Low identity was analyzed separately as the complement of the calibrated identity set.

At a frequency cutoff of 3, every row in the low-identity truth table met the frequency requirement. No logical remainders were therefore available for counterfactual simplification. The intermediate and parsimonious solutions were identical, and the directional expectations did not affect the low-identity configurations. Core and peripheral conditions were identified by comparing the two solutions: conditions appearing in both were classified as core, whereas those appearing only in the intermediate solution were classified as peripheral.

The baseline thresholds were selected by considering methodological guidance, the distinct analytical roles of the two outcome analyses, and the balance between consistency and empirical coverage. For large-N fsQCA, a frequency cutoff of 3 or higher is commonly considered, whereas a PRI cutoff of 0.50 represents the lower acceptable boundary (Greckhamer et al., 2018; Cangialosi, 2023). A more conservative frequency cutoff of 5 and PRI cutoff of 0.65 were used for the focal high-identity analysis, whereas the complementary low-identity analysis used a frequency cutoff of 3 and PRI cutoff of 0.50 as an exploratory specification. A consistency cutoff of 0.80 was used for both outcomes. As reported in the robustness analysis, stricter PRI and frequency settings either reduced empirical coverage or changed the solution composition or core-peripheral assignments. The baseline settings were therefore retained as analytically defensible specifications rather than being treated as uniquely optimal thresholds. The sufficiency analysis identified six configurations associated with high identity and four associated with low identity (Table 12). For high identity, solution consistency was 0.797 and coverage was 0.781. For low identity, solution consistency was 0.834 and coverage was 0.474. The consistency cutoff applies to individual truth-table rows, whereas solution consistency is calculated for the union of the selected configurations. Raising the row-level cutoff therefore does not necessarily produce an overall solution consistency above the same value. Because the high-identity solution consistency remained slightly below 0.80, these configurations should be interpreted as exploratory, sample-specific patterns.

**TABLE 12:** Configurations associated with high and low heritage-related cultural identity.

Antecedent conditions	Configurations for high heritage-related cultural identity						Configurations for low heritage-related cultural identity			
	H1	H2	H3	H4	H5	H6	L1	L2	L3	L4
Web aesthetics	⊗	•	●	●		●		⊗	⊗	⊗
Social media marketing		●	•		●		⊗		⊗	⊗
Cultural heritage understanding	●	●		•	●		⊗	⊗		⊗
Telepresence Scale	●					•	⊗	⊗	⊗	
Social Presence Scale			●	●	•	●	⊗	⊗	⊗	⊗
Raw coverage	0.37	0.55	0.54	0.54	0.55	0.54	0.33	0.33	0.32	0.30
Number of cases	73	111	110	117	130	116	34	32	34	30
Unique coverage	0.02	0.05	0.02	0.01	0.02	0.02	0.06	0.06	0.05	0.03
Consistency	0.89	0.85	0.83	0.86	0.86	0.87	0.86	0.86	0.89	0.89
Overall coverage	% 0.781						% 0.474			
Overall consistency	% 0.797						% 0.834			

Large filled circles (●) indicate the presence of core conditions, while small filled circles (•) indicate the presence of peripheral conditions. Large crossed circles (⊗) indicate the absence of core conditions, whereas small crossed circles indicate the absence of peripheral conditions. Blank spaces indicate that the corresponding antecedent condition may be either present or absent and is therefore irrelevant to the configuration (‘don’t care’).

The six configurations associated with high heritage-related cultural identity can be descriptively grouped into three configuration patterns based on their core conditions. The cognition–immersion pattern (H1) combines the core presence of cultural heritage understanding and telepresence with the core absence of website aesthetics. The cognition–social media pattern (H2 and H5) combines the core presence of cultural heritage understanding and social media marketing, accompanied by the peripheral presence of website aesthetics in H2 and social presence in H5. The aesthetics–social presence pattern (H3, H4, and H6) combines the core presence of website aesthetics and social presence, with social media marketing, cultural heritage understanding, and telepresence appearing as peripheral conditions. These labels are used only as descriptive shorthand for the observed, sample-specific configurations and should not be interpreted as stable user categories or generalizable taxonomies.

The configurations associated with low heritage-related cultural identity likewise indicate that low membership reflects combinations of conditions rather than a single deficient feature. Across the solutions, the absence of cognitive or experiential elements–including cultural heritage understanding, telepresence, or social presence–co-occurred with low heritage-related cultural identity in different combinations. This configurational asymmetry reinforces the distinction between technological exposure and psychologically meaningful media experience.

#### 5.2.4 Robustness test

A robustness check examined the stability of the fsQCA configurations under alternative analytical settings. The truth-table consistency cutoff was increased from 0.80 to 0.85 while the calibrated data, frequency cutoffs, PRI cutoffs, and directional expectations were held constant. The same six high-identity and four low-identity configurations were identified, with unchanged core-condition assignments. Solution consistency and coverage were identical at both cutoffs: 0.797 and 0.781 for high heritage-related cultural identity, and 0.834 and 0.474 for low heritage-related cultural identity, respectively (Supplementary Table 2). The selected truth-table rows already exceeded 0.85, explaining why the higher cutoff did not change the solutions.

Consistent with research that evaluates outcome-specific PRI thresholds through sensitivity comparisons (Douglas and Venugopal, 2025), we examined the sensitivity of the low-identity solution to the PRI cutoff while holding the frequency cutoff at 3 and the consistency cutoff at 0.80. For low heritage-related cultural identity, the baseline PRI cutoff was 0.50. We therefore compared this baseline specification with a stricter cutoff of 0.70. The stricter specification yielded one configuration with solution consistency of 0.902 and coverage of 0.270, compared with four configurations, consistency of 0.834, and coverage of 0.474 under the baseline 0.50 specification. The comparison shows that the stricter PRI cutoff increased consistency but substantially reduced coverage and changed the configuration composition, indicating sensitivity to the PRI specification.

Additional checks also identified sensitivity in the high-identity analysis and in core-condition assignments. Raising the high-identity PRI cutoff from 0.65 to 0.70 reduced the number of configurations from six to four, with solution consistency of 0.824 and coverage of 0.723. Increasing the frequency cutoff from 5 to 6 for high identity yielded seven configurations, with consistency of 0.792 and coverage of 0.787. Increasing the low-identity frequency cutoff from 3 to 5 retained the four intermediate configurations and their aggregate fit, but changed the parsimonious solution and the core–peripheral assignments (Supplementary Table 2). The results therefore support stability in the consistency-cutoff comparison, rather than invariance across analytical settings.

## 6 Discussion

By combining partial least squares structural equation modeling (PLS-SEM) with fuzzy-set qualitative comparative analysis (fsQCA), this study examined how networked communication and interface cues in the Intelligent National Digital Museum were associated with heritage-related cultural identity among Generation Z college students in China through cognitive, spatial, and social psychological states. Overall, the findings support a multipath media-psychological process rather than attributing heritage-related cultural identity to media exposure or any single technological feature. Social media marketing and website aesthetics were associated with cultural heritage understanding, telepresence, and social presence, which were in turn associated with cultural pride, perceived importance of preservation, personal connection, and emotional attachment. The indirect and configurational findings further indicate that the psychological relevance of media cues is expressed both through theory-specified associations involving meaning-making and presence and through heterogeneous combinations of conditions.

### 6.1 Heritage-related cultural identity is grounded in complementary cognitive and presence-based experiences

Among the three direct predictors of heritage-related cultural identity, telepresence had the relatively largest standardized coefficient, followed by cultural heritage understanding and social presence. The differences among the three coefficients were not substantial. The findings therefore support a set of complementary psychological mechanisms rather than the dominance of a single factor. Accordingly, the relatively larger coefficient for telepresence should not be interpreted as evidence that it substantively dominates cultural heritage understanding or social presence. The importance of telepresence is consistent with a fundamental proposition of presence theory: a remote environment acquires psychological reality when users experience a subjective sense of “being there” (Steuer, 1992; Wirth et al., 2007). In digital heritage contexts, this sense of spatial proximity may reduce the psychological distance between users and historical places, making abstract cultural symbols more concrete and more readily connected with the self. This interpretation also resonates with research on place attachment and place identity concerning spatial meaning, self-extension, and emotional connection (Droseltis and Vignoles, 2010; Scannell and Gifford, 2010).

Cultural heritage understanding and social presence also showed positive associations with heritage-related cultural identity. The former provides a cognitive basis for interpreting cultural symbols, historical narratives, and values, whereas the latter captures the perceived psychological presence of communication partners during interactions associated with the digital museum. Collective identification involves positive evaluation, perceived importance, connectedness, satisfaction, and emotional investment in addition to group categorization (Ashmore et al., 2004; Leach et al., 2008). From a media-psychological perspective, these findings suggest that cultural understanding and subjective spatial or social presence are relevant psychological processes linking perceived media cues with self-relevant cultural connections. The study thereby extends the response component of SOR beyond adoption, visitation, or recommendation intentions to an identity-related cognitive–affective outcome.

### 6.2 Networked communication and interface cues perform distinct but complementary psychological functions

Among the three paths originating from social media marketing, its association with cultural heritage understanding was the strongest. In a digital museum context, this pattern supports interpreting ‘social media marketing’ as a set of networked cultural communication cues rather than merely promotional intensity. Interactivity, customization, trend-oriented presentation, and narrative communication may help users organize fragmented information into understandable cultural meanings. Narrative-transportation research indicates that attention, imagination, and emotional involvement enable mediated narratives to produce psychological consequences (Green and Brock, 2000), while cultural-cognition research suggests that cultural cues become relevant to meaning construction when they activate and organize knowledge structures (Hong et al., 2000; Oyserman, 2011). The finding is consistent with Luo et al. (2026) evidence that museum social media marketing activities enhance social presence and complements Mele et al. (2021), who showed that cultural tourism communication on Instagram is culturally patterned and needs to be adapted to different audiences. The present study further suggests that the media-psychological relevance of communication affordances lies in helping cultural content move from being seen to becoming understandable and identity-relevant.

Website aesthetics displayed a more experience-oriented pattern. Its associations with cultural heritage understanding and telepresence were similar in magnitude, and both were numerically larger than its association with social presence. Moreover, in the primary model, the indirect association between website aesthetics and heritage-related cultural identity through telepresence was numerically the largest of the three specific indirect associations originating from website aesthetics. This relatively strong association should not be interpreted as conceptual overlap between website aesthetics and telepresence. The immersion dimension of website aesthetics reflects users’ perception of the interface’s capacity to sustain attentional absorption, whereas telepresence refers to the distinct spatial experience of feeling located within the represented environment. Thus, the two constructs are theoretically proximal but conceptually distinct, with the former representing a perceived media-environment attribute and the latter an internal spatial-experiential state. This distinction is also consistent with the measurement results, which supported discriminant validity between WA and telepresence. Research on processing fluency and aesthetic processing suggests that unified and clearly organized structures can reduce processing demands, whereas novelty and moderate complexity can sustain exploration and elaborative processing (Reber et al., 2004; Leder et al., 2004; Graf and Landwehr, 2015). Evidence from an adjacent digital learning context also links personalized feedback with lower cognitive load and perceived usefulness and enjoyment with greater learning engagement (Li and Wang, 2026). Meta-analytic evidence concerning immersive technologies further indicates that media affordances can enhance users’ overall sense of presence (Cummings and Bailenson, 2016). Xia et al. (2024) showed that website-aesthetics dimensions predicted perceived restoration, which was subsequently associated with place attachment in digital art exhibitions. The present findings further suggest that website aesthetics may be associated with heritage-related cultural identity through the spatial experience of telepresence. Social media marketing and website aesthetics should therefore not be treated as interchangeable technological indicators: the former functions more closely as a communication gateway to cultural meaning, whereas the latter functions more closely as an experiential gateway to cultural space.

Because the stimulus, organism, and response constructs were all assessed through the same retrospective self-report survey, the observed associations may partly reflect shared evaluative or recall processes in addition to the theory-specified relationships. The SOR structure should therefore be interpreted as a theory-guided organization of perceived associations rather than as evidence that objectively measured environmental stimuli produced the observed psychological states.

### 6.3 Cultural heritage understanding functions as a cognitive hub between information processing and presence

Cultural heritage understanding was not only directly associated with heritage-related cultural identity but also indirectly associated with it through telepresence and social presence. The two indirect effects were similar in magnitude, suggesting that spatial and social experiences constitute two parallel pathways through which cultural meanings become connected with identity. Spatial presence theory proposes that users must first construct a coherent mental model of a mediated space before they can adopt that space as their primary spatial frame of reference (Wirth et al., 2007). In a digital museum, understanding historical contexts, symbolic relationships, and narrative structures may make the represented cultural space more coherent and easier to enter psychologically. At the same time, when the actors, social relationships, and shared values underlying cultural artifacts are understood, cultural content may shift from being a display of objects to becoming a representation of a social community, thereby strengthening social presence (Biocca et al., 2003).

In the primary model, all four sequential indirect associations were statistically significant, although their magnitudes were small. When direct paths from SMM and WA to heritage-related cultural identity were added, the two WA-originating sequential associations decreased to β = 0.007 and no longer met the conventional 0.05 significance threshold (WA → CHU → TPS → CI: *p* = 0.052; WA → CHU → SPS → CI: *p* = 0.061). These findings are consistent with the theory-specified sequence linking cultural heritage understanding, presence, and heritage-related cultural identity, but the statistical support for the two WA-originating sequences was sensitive to model specification. Because the data are cross-sectional, these sequential associations do not establish temporal ordering. Longitudinal or experimental studies are needed to examine whether the proposed sequence occurs over time.

### 6.4 Configurational heterogeneity, equifinality, and asymmetry in heritage-related cultural identity

The fsQCA results showed that no single media-environment or psychological condition was universally necessary for high heritage-related cultural identity. The six high-identity configurations could be summarized as cognition–immersion, cognition–social media, and aesthetics–social presence patterns. These results indicate configurational heterogeneity: membership in the set representing high heritage-related cultural identity was associated with multiple alternative combinations of conditions rather than with a single uniform configuration. Here, heterogeneity refers specifically to variation in sufficient condition combinations within the sample, rather than to evidence that different individuals necessarily follow distinct psychological trajectories over time. The configurational results should therefore be interpreted as set-theoretic, sample-specific patterns rather than as distinct developmental pathways or stable user types. Given the high-identity solution consistency of 0.797 and the sensitivity observed in the PRI and frequency checks, these configurations should be interpreted as exploratory, sample-specific patterns. Their detailed composition and core-condition assignments depend on analytical choices and require further verification.

Viewed together, the configurations suggest that the cognitive, spatial, social, and media-environment conditions may relate to one another through different forms of conjunctural dependence. The cognition–immersion configuration is consistent with a compensatory pattern, because strong cultural heritage understanding and telepresence can coexist with high heritage-related cultural identity even when website aesthetics is core-absent. The cognition–social media configurations suggest complementarity between networked cultural communication and cultural meaning-making, whereas the aesthetics–social presence configurations suggest complementarity between interface-based experience and social presence. Across these configurations, the absence of a condition from one high-identity solution while other conditions remain present also points to partial substitutability rather than universal necessity. These patterns should not be interpreted as causal substitution among variables, but as sample-specific evidence that different combinations of conditions may play partially overlapping roles in relation to the same outcome.

The cognition–immersion pattern was particularly noteworthy. Even when website aesthetics was a core absent condition, the core presence of cultural heritage understanding and telepresence still co-occurred with high heritage-related cultural identity. This finding does not mean that website design is unimportant. Instead, it indicates that aesthetic strength is not a necessary prerequisite in every pathway to high heritage-related cultural identity. When cultural content is deeply understood and accompanied by a strong sense of spatial presence, limitations in interface aesthetics may be compensated for to some extent. Conversely, the aesthetics–social presence pattern indicates that the combination of website experience and social presence can constitute an alternative pathway to high heritage-related cultural identity. The configurations associated with low heritage-related cultural identity were not simple mirror images of those associated with high heritage-related cultural identity, suggesting that fostering higher levels of heritage-related cultural identity and reducing the conditions associated with lower levels of heritage-related cultural identity require attention to different combinations of conditions. This asymmetry helps explain why a single strategy of “improving the website” or “strengthening communication” is unlikely to produce identical outcomes for all users.

Importantly, these configurational findings qualify the interpretation of the positive average associations identified by PLS-SEM. A condition may show a positive net association at the aggregate level without being universally necessary in every configuration associated with high heritage-related cultural identity. For example, website aesthetics was positively associated with the organismic states in the structural model, yet one high-identity configuration combined the core presence of cultural heritage understanding and telepresence with the core absence of website aesthetics. Thus, the PLS-SEM results should be interpreted as average tendencies across the sample rather than as evidence of a single media-psychological architecture that must be instantiated similarly across cases. The fsQCA results therefore add a configurational boundary to the average SOR relationships by showing that different combinations of cognitive, spatial, social, and media-environment conditions can be associated with the same identity-related outcome.

## 7 Theoretical and practical implications

### 7.1 Theoretical implications

First, this study extends SOR-based media-effects research by positioning heritage-related cultural identity, rather than technology adoption or subsequent behavior, as the focal response. Existing digital-museum studies often model cultural identity as an antecedent or mediator of visitation and electronic word of mouth (Meng and Dolah, 2025; Zhang et al., 2026). By showing that perceived media cues are associated with identity through cognitive and presence-based states, the study identifies meaning-making and subjective presence as media-psychological processes linking digital heritage encounters with self-relevant cultural evaluation.

Second, the study refines both the stimulus and organism components of SOR. It distinguishes networked communication cues from interface-based cues and, within the organism, separates cultural understanding from spatial and social presence. This structure explains why digital museums should not be treated as homogeneous technical environments: communication and interface affordances can converge on the same identity-related response through partially overlapping cognitive, spatial, and social pathways. Within the theory-specified model, cultural heritage understanding is positioned before both forms of presence, providing a parsimonious account of how interpreted cultural meaning is associated with spatial and social experience without implying an empirically established temporal sequence. It does not assume that any single mechanism is universally dominant.

Third, the combined findings introduce configurational heterogeneity into the SOR explanation of heritage-related cultural identity. PLS-SEM identifies positive average associations among media-environment cues, cognitive and presence-based states, and heritage-related cultural identity, but the fsQCA results show that these associations do not imply a single uniform architecture across cases. No single condition was universally necessary for high heritage-related cultural identity, and alternative configurations were associated with the same outcome. The combined evidence therefore distinguishes average importance from configurational necessity: a condition may contribute positively at the aggregate level while being absent from a particular high-identity configuration. This places an important boundary on single-path and technology-determinist interpretations of SOR and suggests that cognitive, spatial, social, and media-environment conditions may operate through partially complementary, compensatory, or substitutable configurations. In particular, the core absence of website aesthetics in one high-identity configuration suggests that technologically favorable interface conditions are not universally required when cultural heritage understanding and spatial presence are both present as core conditions. SOR therefore remains useful as an organizing framework for average stimulus–organism–response associations, while the configurational results show that it should not be read as specifying one invariant architecture through which all cases reach the same identity-related outcome.

### 7.2 Practical implications

The findings suggest that digital museum performance should be evaluated not only in terms of traffic, reach, or interface novelty, but also by whether users understand cultural content and experience it as spatially and socially present. In the primary model, telepresence, cultural heritage understanding, and social presence showed associations of similar magnitude with heritage-related cultural identity (β = 0.234, 0.210, and 0.192, respectively). This pattern suggests that managers should balance cultural interpretation, immersive spatial presentation, and socially engaging narratives rather than concentrating resources on a single technology. Official social media should therefore move beyond promotion toward meaning-making by providing concise historical context, artifact provenance, thematic storytelling, expert interpretation, and guided interaction. Such content should remain accessible without sacrificing historical accuracy for online visibility.

Website and exhibition design should prioritize “comprehensible immersion.” Clear visual hierarchies, coherent navigation, perceptible spatial relations, controllable three-dimensional views, and contextual annotations are more valuable than decorative complexity when they help users construct meaningful representations of cultural settings. The configurational findings further suggest that no standardized experience will be equally effective for all users. Platforms may use these sample-specific configuration patterns as exploratory reference points when considering flexible combinations of cognition, communication, aesthetics, and presence-oriented design features. They should not be treated as fixed user segments or stable design archetypes. When resources are limited, content accuracy, accessibility, loading performance, and system stability should take priority over costly visual effects.

For youth engagement, digital museums can connect online exhibitions with university courses, campus activities, and offline visits. Evaluation indicators should extend beyond clicks and viewing time to include cultural understanding, presence, identity-related connection, and subsequent cultural participation. Pre–post assessments and anonymized behavioral data can support continuous improvement. However, because this study did not examine algorithmic recommendation or identity inference, its findings do not justify intensive user profiling. Any personalization should be implemented cautiously, with safeguards for privacy, cultural accuracy, and the avoidance of stereotypical representation.

## 8 Limitations and future research directions

This study has several limitations. First, its cross-sectional, self-report design cannot establish temporal order or causal relationships. In addition, all focal constructs were assessed retrospectively from the same respondents within a single survey. This common-source design may introduce shared evaluative or recall variance and potential common-method bias, particularly because SMM and WA represent perceived evaluations of prior media experiences rather than independently measured environmental characteristics. The primary theory-specified model did not include direct paths from social media marketing or website aesthetics to heritage-related cultural identity. An alternative robustness specification including these paths was therefore estimated. Both direct associations were statistically significant, while most of the principal indirect pathways remained stable. However, the two sequential associations originating from WA did not reach the 0.05 significance level in the alternative model, so these specific results were less robust. The findings are therefore more consistent with the coexistence of direct and indirect associations than with a full-mediation interpretation. Because the data are cross-sectional, however, neither the primary nor the alternative specification establishes temporal or causal mediation. Future longitudinal, cross-lagged, and experimental studies should compare direct and alternative sequences, including whether presence precedes understanding or whether pre-existing heritage-related cultural identity influences platform evaluations. Future research could further combine self-reports with objective interface indicators, platform-use records, experimentally manipulated media features, or temporally separated measurements to reduce reliance on common-source retrospective assessments.

Second, the sample comprised 425 Chinese Generation Z college students with prior experience of a single digital museum platform. Participants evaluated their overall previous experience rather than a standardized exhibition. Because detailed information on usage frequency, duration, recency, and specific exhibition experience was not systematically collected, these sources of heterogeneity could not be fully controlled in the present analysis. These characteristics limit the generalizability of the findings. Replication across platforms, exhibitions, age groups, levels of digital competence, and cultural contexts–preferably using multilevel designs–is therefore required.

Third, the primary model explained 22.2% of the variance in heritage-related cultural identity, indicating meaningful but still modest and incomplete explanatory coverage. Future research could incorporate pre-existing cultural identity, heritage familiarity, perceived authenticity, narrative quality, institutional trust, cultural socialization, need for cognition, immersive propensity, and digital literacy. Because social media marketing and website aesthetics were modeled as reflective–reflective higher-order constructs, the present analysis also cannot isolate the contribution of each lower-order dimension. Theory-driven decomposition and experimental manipulation could clarify their independent and combined effects.

Finally, the fsQCA findings remain dependent on the sample, calibration decisions, and truth-table parameters. Increasing the consistency cutoff from 0.80 to 0.85 left the configurations and core-condition assignments unchanged, whereas changes in the PRI and frequency cutoffs affected the results. Together with the high-identity solution consistency of 0.797, this sensitivity limits the strength of the configurational conclusions. Future studies should examine these patterns using independently justified analytical settings and independent samples. The study also did not examine identity expression, behavioral personalization, or algorithmic feedback loops. Future research could integrate longitudinal behavioral traces, experiments, and algorithm audits while systematically addressing privacy, bias, and culturally stereotypical personalization.

## Data Availability

The raw data supporting the conclusions of this article will be made available by the authors, without undue reservation.
