# Supplementary material for: Psychological pathways linking digital museum environments to heritage-related cultural identity among generation Z college students in China
Source: Front Psychol. 2026 Oct 27;17:1979445. doi: 10.3389/fpsyg.2026.1979445 (PMC13748410; doi:10.3389/fpsyg.2026.1979445)
Supplement: Supplementary file 1 [file Supplementary_file_1.docx]

**Supplementary Table 1. Retained Measurement Items and Source Scales**

| **Construct** | **Items** | **Source** |
| --- | --- | --- |
| **Website Aesthetics** |  |  |
| Perception Unity | Q1: The exhibits displayed are consistent with the theme of the digital museum.  Q2: The text and colors on the digital museum website are well coordinated.  Q3: The overall atmosphere of the digital museum is harmonious and consistent. | Jiang et al., 2016; Vashisht &  Chauhan, 2017; Tcha-Tokey et al.,  2016; Xia et al., 2024 |
| Perceived Novelty | Q1: I have never seen a digital museum like this before.  Q2: The integration of new technologies and art exhibitions in this digital museum is highly innovative.  Q3: This digital museum is novel to me. |  |
| Perception Interactivity | Q1: My actions determine the type of experience I obtain in the digital museum.  Q2: I actively participate in interactive experiences in the online digital museum.  Q3: I can freely choose the content I want to explore. |  |
| Perception Immersion | Q1: I am completely focused on this digital museum.  Q2: I lose track of time while exploring this digital museum.  Q3: This digital museum gives me a strong sense of immersion. |  |
| **Social Media Marketing** |  |  |
| Entertainment | Q1: Using the digital museum’s social media platform is enjoyable.  Q2: The content presented on the digital museum’s social media platform is interesting.  Q3: The digital museum’s social media platform is entertaining. | Kim & Ko, 2012; Liu et al., 2023; Çil et al., 2023 |
| Interaction | Q1: The digital museum’s social media platform enables information sharing.  Q2: I can interact with others through the digital museum’s social media platform.  Q3: I can easily express my opinions through the digital museum’s social media platform. |  |
| Trendiness | Q1: The content presented on the digital museum’s social media platform provides up-to-date information.  Q2: Using the digital museum’s social media platform is trendy.  Q3: The content presented on the digital museum’s social media platform reflects current trends. |  |
| Customization | Q1: The digital museum’s social media platform provides customized information search.  Q2: The digital museum’s social media platform offers personalized services.  Q3: The digital museum’s social media platform provides useful information that meets my needs. |  |
| **Cultural Heritage** **Understanding** | Q1: I fully understand the history and culture presented in the digital museum.  Q2: I can identify the historical and cultural elements presented in the digital museum.  Q3: I can gain a deeper understanding of the cultural characteristics presented in the digital museum. | Wang & Meng, 2024 |
| **Telepresence Scale** | Q1: When using the digital museum, I often forget my physical surroundings.  Q2: When participating in the digital museum’s virtual exhibitions, I feel as if I am experiencing an immersive cultural journey.  Q3: When participating in the digital museum’s virtual exhibitions, I feel as if I am in the cultural world created by the virtual museum. | Krasonikolakis et al., 2018; Zhu et al., 2025; Alalwan et al., 2025 |
| **Social Presence Scale** | Q1: When engaging in asynchronous communication in the digital museum’s virtual exhibitions, I can imagine my communication partners.  Q2: When engaging in real-time communication in the digital museum’s virtual exhibitions, I feel as if I am communicating with real people rather than anonymous others.  Q3: Real-time communication in the digital museum’s virtual exhibitions feels almost like face-to-face interaction. | Kreijns et al., 2011; Alsayer & Lowenthal, 2025 |
| **Heritage-related cultural identity** | Q1: I feel proud of the history and culture presented in the digital museum.  Q2: I believe that preserving the history and culture presented in the digital museum is very important.  Q3: Being connected to the history and culture presented in the digital museum is important to me.  Q4: I feel emotionally attached to the history and culture presented in the digital museum. | Cleveland et al., 2016; Zhang et al., 2019; Song et al., 2025 |

**Supplementary Table 2. Sensitivity of the high- and low-identity configurations to consistency, PRI, and frequency cutoffs**

| **Outcome** | **Specification** | **Freq.** | **Cons. cutoff** | **PRI** | **Config. count** | **Solution cons.** | **Solution cov.** |
| --- | --- | --- | --- | --- | --- | --- | --- |
| High | Baseline | 5 | 0.80 | 0.65 | 6 | 0.797 | 0.781 |
| High | Consistency .85 | 5 | 0.85 | 0.65 | 6 | 0.797 | 0.781 |
| High | PRI .70 | 5 | 0.80 | 0.70 | 4 | 0.824 | 0.723 |
| High | Higher frequency | 6 | 0.80 | 0.65 | 7 | 0.792 | 0.787 |
| Low | Baseline | 3 | 0.80 | 0.50 | 4 | 0.834 | 0.474 |
| Low | Consistency .85 | 3 | 0.85 | 0.50 | 4 | 0.834 | 0.474 |
| Low | PRI .70 | 3 | 0.80 | 0.70 | 1 | 0.902 | 0.270 |
| Low | Higher frequency | 5 | 0.80 | 0.50 | 4 | 0.834 | 0.474 |

Note. Configuration counts refer to intermediate solutions. Raising the consistency cutoff from 0.80 to 0.85 retained the configurations and core–peripheral assignments reported in Table 12 for both outcomes. Raising the low-identity frequency cutoff from 3 to 5 retained the four intermediate configurations, but the absence of CHU in L1, TPS in L2, WA in L3, and SMM in L4 changed from core to peripheral. All other condition assignments within these four configurations remained unchanged.
